# *Piper sarmentosum* Roxb. methanolic extract prevents stress-induced gastric ulcer by modulating oxidative stress and inflammation

**DOI:** 10.3389/fphar.2022.971443

**Published:** 2023-01-12

**Authors:** Muhamad Nurul Akmal, Ibrahim Abdel Aziz, Mohd Fahami Nur Azlina

**Affiliations:** ^1^ Department of Pharmacology, Faculty of Medicine, UKM Medical Centre, Universiti Kebangsaan Malaysia, Kuala Lumpur, Malaysia; ^2^ Department of Pharmacology and Toxicology, Faculty of Medicine, Umm Al-Qura University, Makkah, Saudi Arabia

**Keywords:** *Piper sarmentosum*, malondialdehyde, gastric ulcer, nitric oxide, iNOS, IL-1β, TNF-α, IL-6

## Abstract

This study investigated the gastroprotective effect of *Piper sarmentosum* (PS) on stress-induced gastric ulcers in rats by measuring its effect on oxidative stress, gastric mucosal nitric oxide (NO), and inflammatory biomarkers. Twenty-eight male Wistar rats were randomly divided into four groups; two control groups (non-stress and stress) and two treated groups supplemented with either methanolic PS extract (500 mg/kg body weight) or omeprazole (OMZ; 20 mg/kg) orally. After 28 days of treatment, the stress control, PS, and OMZ groups were subjected to water-immersion restrain stress (WIRS) for 3.5 h. Gastric tissue malondialdehyde (MDA), NO, superoxide dismutase (SOD), inducible NO synthase (iNOS), SOD mRNA, tumor necrosis factor (TNF)-α, interleukin (IL)-1β, and IL-6 levels were measured. WIRS significantly increased gastric MDA, NO, and pro-inflammatory cytokine levels compared to the non-stressed control group. PS and omeprazole supplementation significantly reduced WIRS-exposure-induced gastric ulcers and MDA, iNOS, and IL-1β levels. However, only PS reduced NO, TNF-α, and IL-6 levels, which were upregulated in this ulcer model. In conclusion, the gastroprotection afforded by PS is possibly mediated by gastric mucosal NO normalization through reduced iNOS expression and attenuation of inflammatory cytokines. PS showed a greater protective effect than omeprazole in reducing gastric lesions and NO, TNF-α, and IL-6 levels, and iNOS expression.

## 1 Introduction

Upper gastrointestinal bleeding is associated with significant morbidity and mortality. Bleeding peptic ulcers remain the most common cause of acute non-variceal upper gastrointestinal bleeding. This condition is often associated with non-steroidal anti-inflammatory drug use (Atchison et al., 2013), *Helicobacter pylori* infection (Wang et al., 2014), and stress (Nur Azlina et al., 2013; Kudryavtsev et al., 2014). Stress ulcer syndrome can cause mucosal erosions and superficial hemorrhages in critically ill patients or individuals under extreme physiologic stress. The ulcers frequently emerge due to major stressful events, including surgery, trauma, shock, sepsis, and burns. This study used water-immersion restraint stress (WIRS), an established method to induce gastric lesions in rats (Konturek et al., 2008; Kwiecien et al., 2012; Raja Kumar et al., 2019), to create a stress model that mimics stress ulcers in human patients.

Stress is associated with increased oxidative stress due to increased free radical formation. Kwiecien et al. (2012) found that WIRS causes acute inflammatory responses, with interleukin (IL)-1 and tumor necrosis factor-alpha (TNF-α) acting as the primary pro-inflammatory cytokines induced by neutrophil infiltration into the gastric mucosa. Neutrophils produce superoxide anions (O_2_^•-^) that react with cellular membrane lipids, resulting in lipid peroxidation. Malondialdehyde (MDA) and 4-hydroxynonenal are examples of lipid peroxidation end-products (Khoubnasabjafari et al., 2015). Stress will also produce high nitric oxide (NO) levels. NO is catalyzed by inducible NO synthase (iNOS) and acts as a potent cytotoxic oxidant (Lanas, 2008; Nimse and Pal, 2015). Stress also decreases superoxide dismutase (SOD) activity in gastric mucosa, impairing its antioxidative defense mechanism (Kwiecień et al., 2002; Nur Azlina et al., 2013; Patlevič et al., 2016).

*Piper (P.) sarmentosum* Roxb. belongs to the Piperaceae family. It is an about 20 cm tall herb that grows wild in the forest and is commonly found in Southeast Asia (e.g., Malaysia, Cambodia, Philippines, Thailand, and Myanmar). *P. sarmentosum* is a traditional medicinal plant whose leaves are usually eaten raw as *ulam*. For centuries, it has been used to treat wind-cold cough, fever, postpartum foot edema, stomachache, toothache, diabetes, and traumatic injury (Sun et al., 2020). *P. sarmentosum* has anti-inflammatory (Zakaria et al., 2010; Azlina et al., 2019; Salehi et al., 2019) and anti-atherosclerotic (Amran et al., 2010) properties. Methanolic *P. sarmentosum* extract has been shown to have antioxidative activity due to the natural antioxidant superoxide scavenger, naringenin (Subramaniam et al., 2003; Chanwitheesuk et al., 2005). Therefore, this study investigated the protective effects of methanolic *P. sarmentosum* extract against gastric mucosa injury as an alternative to other antioxidants. We used omeprazole as a positive control since it is one of the most widely prescribed drugs to treat gastric ulcers (Hajrezaie et al., 2012; Ketuly et al., 2013).

## 2 Materials and methods

### 2.1 Plant materials

Fresh *P. sarmentosum* leaves were collected from the Forest Research Institute Malaysia (FRIM) reserve forest at Kepong, Selangor, Malaysia, and identified by FRIM’s Medicinal Plant Division. A voucher specimen (FRI 45870) was deposited at FRIM’s Medicinal Plant Division.

### 2.2 Preparation of methanolic *P. sarmentosum* extract

The methanolic extraction procedure was performed at the FRIM laboratory. The leaves were cleaned with tap water and dried at room temperature before being finely chopped. Next, 250 g of leaves were extracted in 2.5 L of methanol. This mixture was heated to 40°C–60°C using a Soxhlet to evapourate the methanol (Sawangjaroen et al., 2004). The paste material produced was kept at 4°C until required. The percentage yield from the crude dried extract was around 10%. The plant extract was analyzed using liquid chromatography-mass spectrometry at the Universiti Kebangsaan Malaysia’s (UKM) Research and Instrumentation Management Center. The active chemical and purity results have been previously reported (Bactiar and Fahami, 2019). Methanol *P. sarmentosum* leaf extracts yielded fifteen compounds extracted and characterized by spectroscopic methods, including didymin, naringenin, methyl piperate, quercetin, beta asarone, brachyamide, amurensin, piperitol, guineensine, hesperidin, rutin, malvidin, and difucol (Table 1). The term *P. sarmentosum* in this study refers to *P. sarmentosum* methanolic extract.

**TABLE 1:** Isolated compound from methanol *P. sarmentosum* leaf extracts.

No	Name of compound	Formula	Molecular weight	Molecular structure
1	Didymin	C_28_H_34_O_14_	594.6	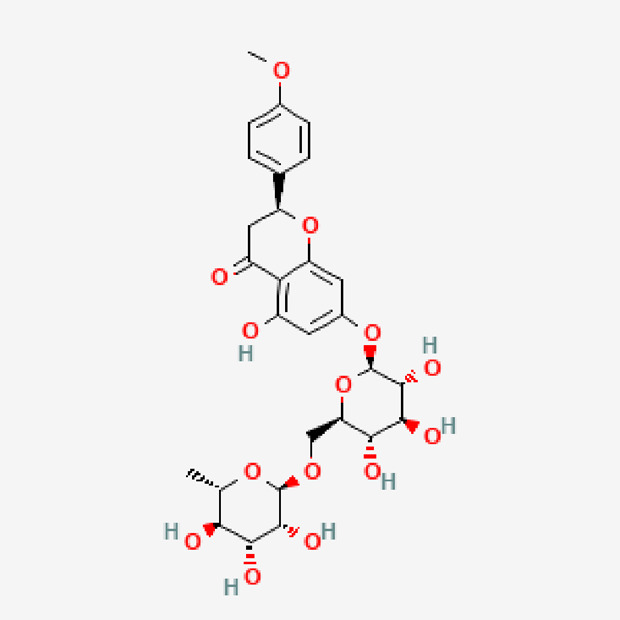
				PubChem Identifier: CID 16760075 URL: https://pubchem.ncbi.nlm.nih.gov/compound/16760075#section=2D-Structure
2	Naringenin	C_15_H_12_O_5_	272.25	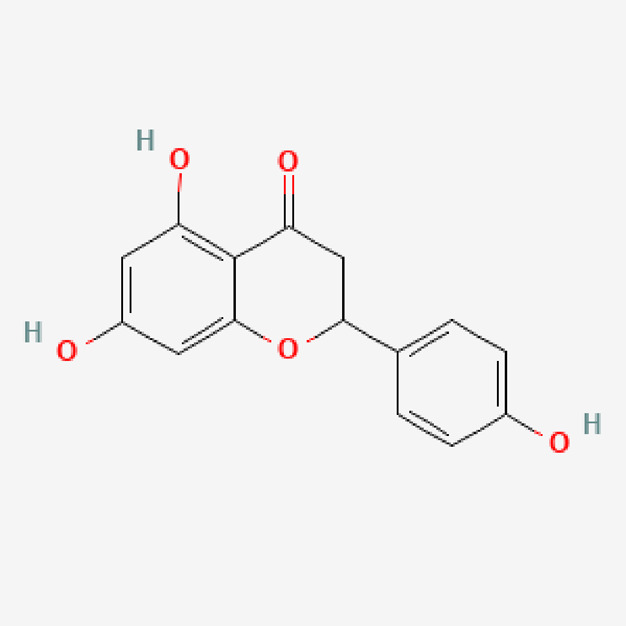
				PubChem Identifier: CID 932 URL: https://pubchem.ncbi.nlm.nih.gov/compound/932#section=2D-Structure
3	Methyl piperate	C_13_H_12_O_4_	232.23	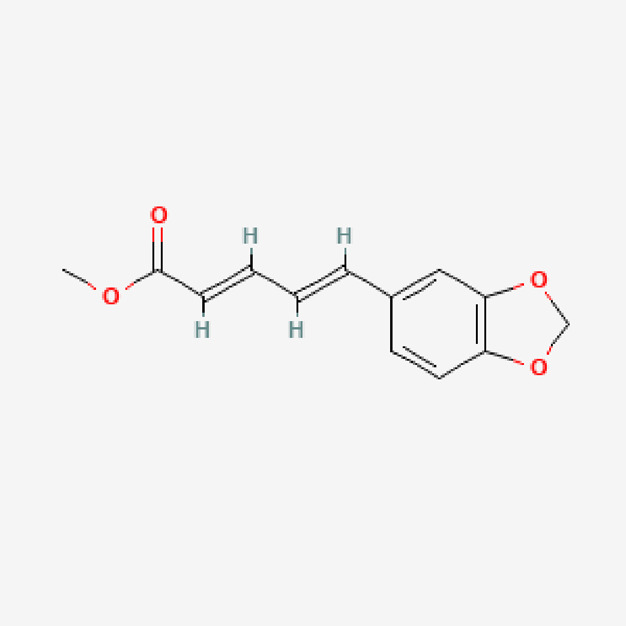
				PubChem Identifier: CID 9921021 URL: https://pubchem.ncbi.nlm.nih.gov/compound/9921021#section=2D-Structure
4	Quercetin	C_15_H_10_O_7_	302.23	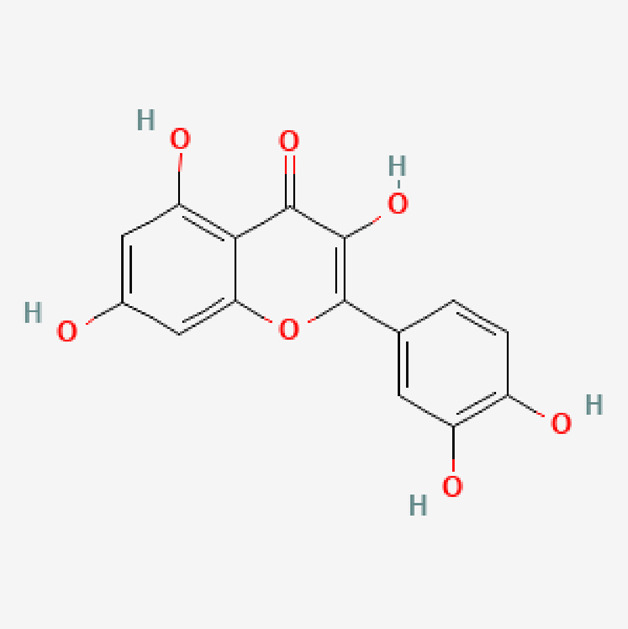
				PubChem Identifier: CID 5280343 URL: https://pubchem.ncbi.nlm.nih.gov/compound/5280343#section=2D-Structure
5	Beta-Asarone	C_12_H_16_O_3_	208.25	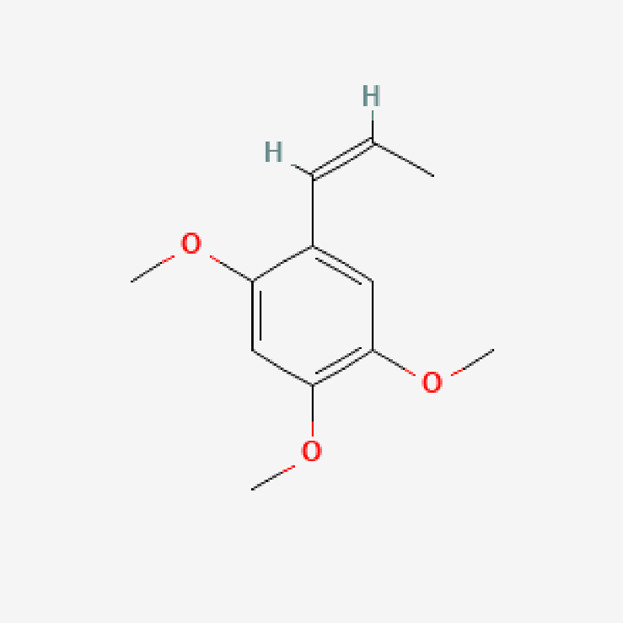
				PubChem Identifier: CID 5281758 URL: https://pubchem.ncbi.nlm.nih.gov/compound/5281758#section=2D-Structure
6	Brachyamide B	C_20_H_25_NO_3_	327.4	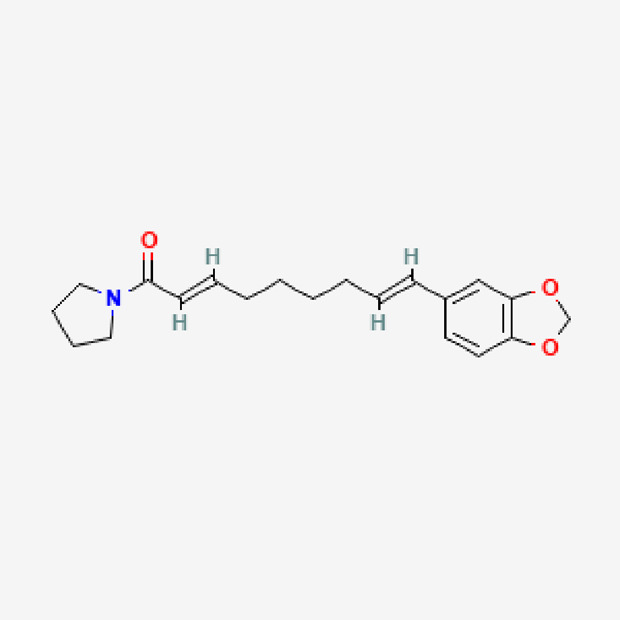
				PubChem Identifier: CID 14162526 URL: https://pubchem.ncbi.nlm.nih.gov/compound/14162526#section=2D-Structure
7	Amurensin	C_26_H_30_O_12_	534.5	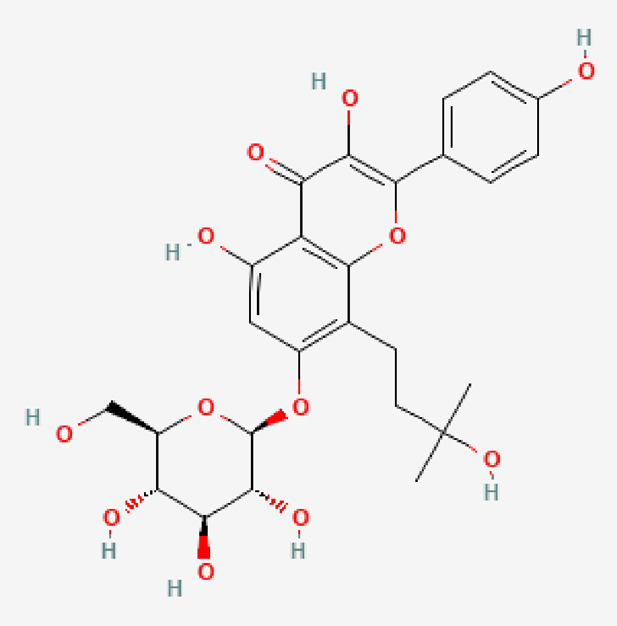
				PubChem Identifier: CID 5318156 URL: https://pubchem.ncbi.nlm.nih.gov/compound/5318156#section=2D-Structure
8	Piperitol	C_20_H_20_O_6_	356.4	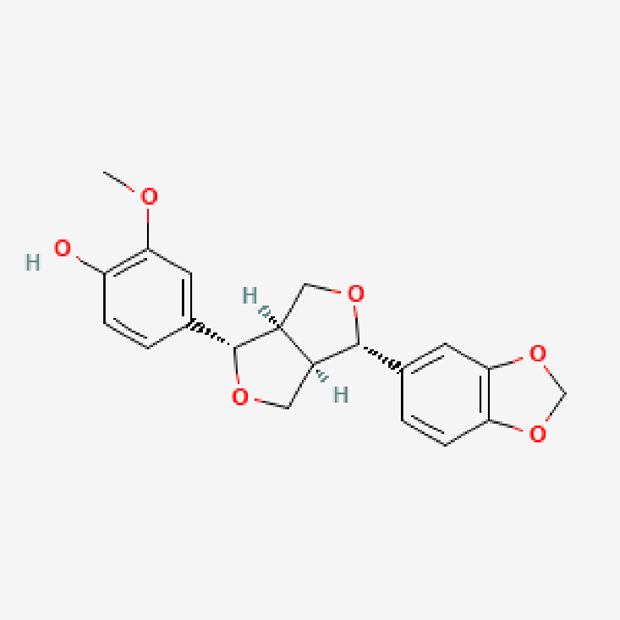
				PubChem Identifier: CID 10247670 URL: https://pubchem.ncbi.nlm.nih.gov/compound/10247670#section=2D-Structure
9	Guineensine	C_24_H_33_NO_3_	383.5	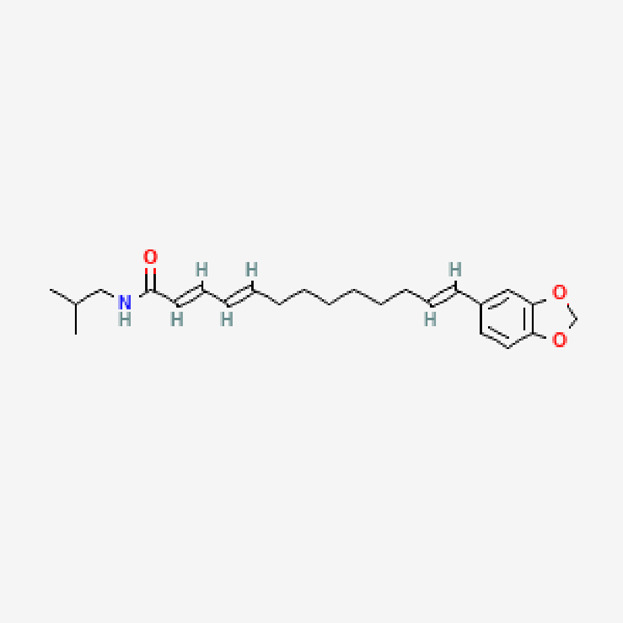
				PubChem Identifier: CID 6442405 URL: https://pubchem.ncbi.nlm.nih.gov/compound/6442405#section=2D-Structure
10	Hesperidin	C_28_H_34_O_15_	610.6	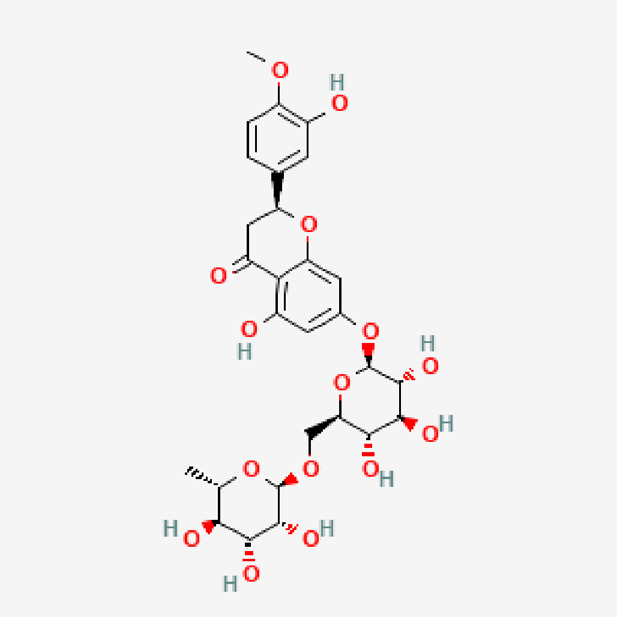
				PubChem Identifier: CID 10621 URL: https://pubchem.ncbi.nlm.nih.gov/compound/10621#section=2D-Structure
11	Rutin	C_27_H_30_O_16_	610.5	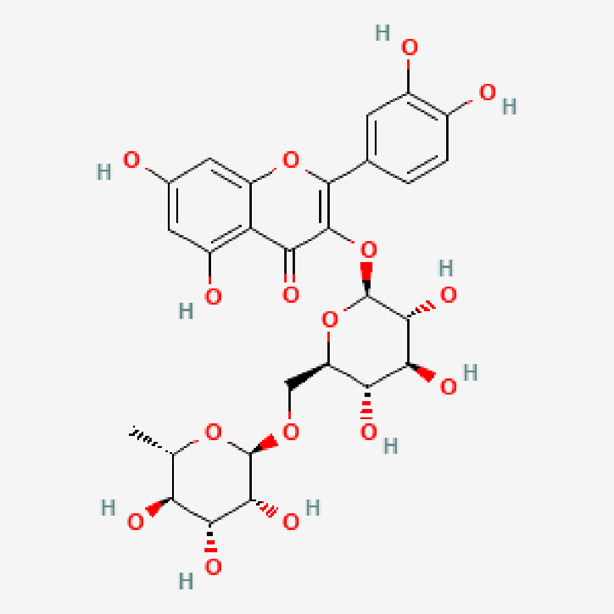
				PubChem Identifier: CID 5280805 URL: https://pubchem.ncbi.nlm.nih.gov/compound/5280805#section=2D-Structure
12	Malvidin	C_17_H_15_O_7_^+^	331.30	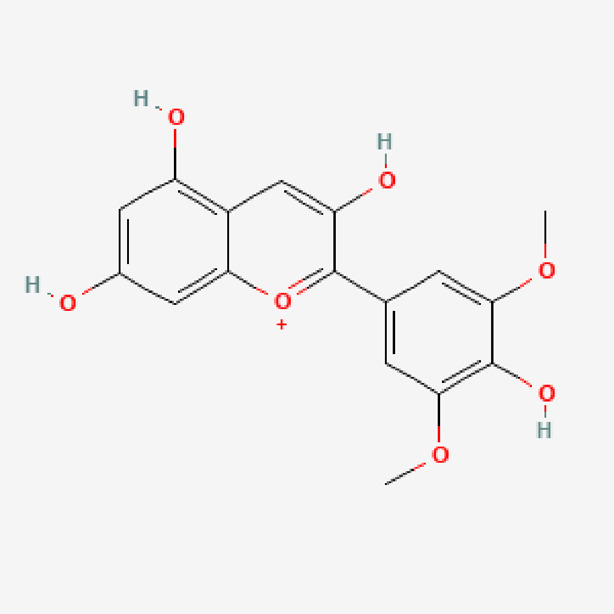
				PubChem Identifier: CID 159287 URL: https://pubchem.ncbi.nlm.nih.gov/compound/159287#section=2D-Structure
13	Difucol	C_12_H_10_O_6_	250.20	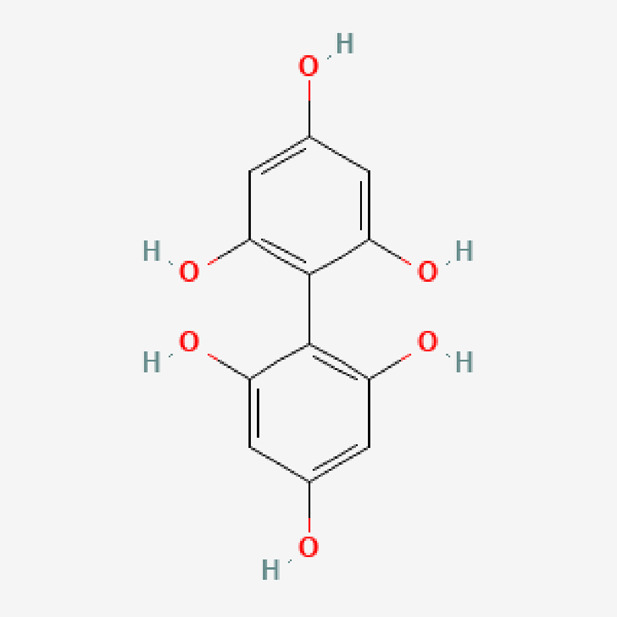
				PubChem Identifier: CID 433697 URL: https://pubchem.ncbi.nlm.nih.gov/compound/433697#section=2D-Structure

### 2.3 Experimental design

Twenty-eight male *Sprague Dawley* rats (obtained from the Animal Unit, Faculty of Medicine, UKM) were divided into four groups: non-stress control, stress control, *P. sarmentosum*-treated, and omeprazole-treated. The non-stress and stress control groups were administered vitamin E-free palm oil *via* oral gavage. Omeprazole and *P. sarmentosum* were diluted in vitamin E-free palm oil as a vehicle. Omeprazole (20 mg/kg body weight) and *P. sarmentosum* extract (500 mg/kg body weight) were administered *via* oral gavage. This *P. sarmentosum* dose was chosen based on our previous study showing a protective *P. sarmentosum* methanolic extract effect on stress-induced gastric lesions (Azlina et al., 2019). Throughout the feeding period, all rats were habituated to handling to reduce their stress-related disturbances. After 28 days, the rats in the stress control, omeprazole-treated, and *P. sarmentosum*-treated groups were exposed to WIRS for 3.5 h. The rats in the non-stress control group were not subjected to any stress. The rats were restrained in a plastic restrainer before being placed individually in a beaker containing room-temperature tap water. The water level was adjusted to the rat’s neck level (Azlina et al., 2015). Then, the rats were sacrificed, and their stomach was isolated to measure gastric lesion index, gastric MDA content, NO level, iNOS mRNA level, SOD activity, SOD mRNA levels, TNF-α, IL-1β, and IL-6 levels. This study was approved by the UKM Animal Ethics Committee (354/2011).

### 2.4 Parameters measurements

#### 2.4.1 Gastric lesion

Gastric lesions were measured using an image analyzer at ×3 magnification. The lesion length in mm was measured at the lesion’s greatest diameter. Each five petechial was equal to a 1 mm lesion. Lesion length was expressed as the lesion index according to a previously described method (Ibrahim et al., 2012).

#### 2.4.2 Gastric MDA levels

Gastric MDA levels were measured using a previously described method (Ledwoz et al., 1986). Briefly, 2.5 mL of a trichloroacetic acid (1.22 mol/L) and hydrochloric acid (.6 mol/L) solution was added to .5 mL of the sample and incubated at room temperature for 15 min. Next, .05 mL of sodium hydroxide was added, and the sample was incubated at 100°C for 30 min before being cooled to room temperature. Then, 4 mL of n-butanol was added, and the sample was vigorously vortex for 3 min. Finally, the sample was centrifuged at 3,000 rpm for 10 min, and the absorbance of the upper layer at 535 nm was measured using a spectrophotometer.

#### 2.4.3 Gastric SOD levels

SOD levels in gastric tissue were measured using Cayman’s SOD Assay Kit (70600; Ann Arbor, MI, United States). This kit uses a tetrazolium salt to detect O_2_^•-^ generated by xanthine oxidase.

#### 2.4.4 NO levels

NO levels in gastric tissue were measured using the Quantichrome NO Assay Kit (D2NO-100; BioAssay Systems; Hayward, CA, United States). NO was oxidized to nitrate and nitrite. Total nitrate and nitrites levels were quantified based on the absorbance at 540 nm measured using an ELISA reader.

#### 2.4.5 SOD and iNOS mRNA levels

SOD and iNOS mRNA levels were measured using the QuantiGene Plex Assay Lit (Genospectra; Fremont, CA, United States). Tissue lysate was added to a well containing a gene-specific probe set and then hybridized overnight at 53°C. Next, wells were washed twice with bDNA wash buffer before being incubated at 46°C with an amplifier and then an alkaline phosphatase-linked label probe, with a wash step in between. Finally, streptavidin phycoerythrin was added, producing a luminescent signal proportional to the target RNA amount that was measured using a Luminex machine (Zhang et al., 2005).

#### 2.4.6 TNF-α, IL-1β, and IL-6 protein quantification assays

TNF-α, IL-1β, and IL-6 levels in gastric tissue were measured using Panomics’ Procarta Cytokine Assay Kit (Affymetrix; Santa Clara, CA, United States) and a Luminex 200 analyzer (Luminex Corporation; Darmstadt, Germany). Procarta Protein Assays use xMAP technology (multi-analyte profiling beads) to enable simultaneous quantitation of multiple protein targets. The xMAP system combines a flow cytometer, fluorescent-dyed microspheres (beads), lasers, and digital signal processing to effectively multiplex up to 100 different assays within a single sample.

### 2.5 Statistical analysis

Statistical analyses were performed using SPSS v.23 (SPSS Inc., Chicago, IL, United States). The normality of each variable’s distribution was assessed using the Shapiro–Wilk test. All results are expressed as the mean ± standard error of the mean (SEM). The significance (*p* < .05) of differences between groups was assessed with an analysis of variance followed by Tukey’s *post hoc* test.

## 3 Results

### 3.1 Gastric lesions

**FIGURE 1:**
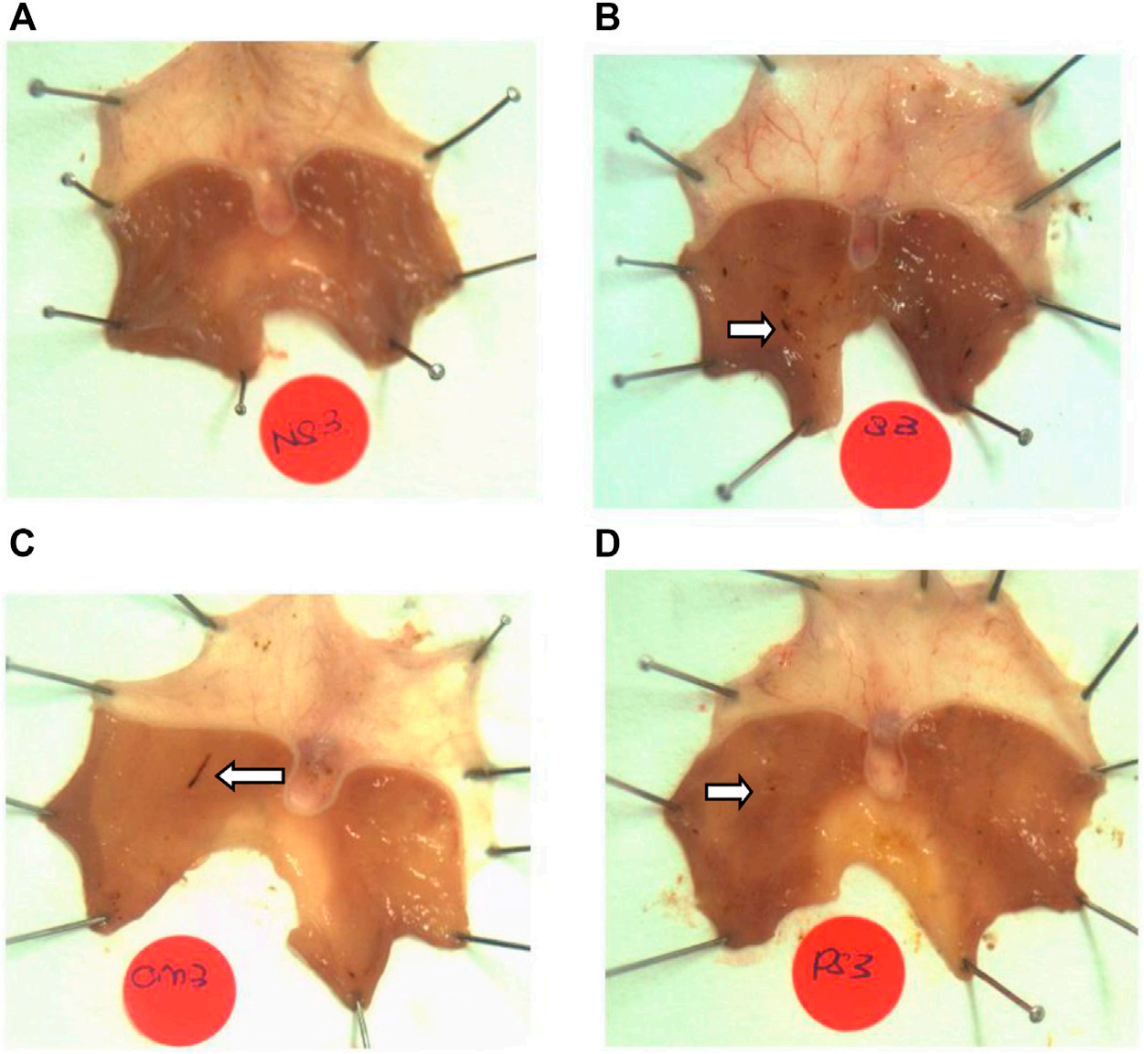
Macroscopic observation of gastric lesions in **(A)** normal rats (no lesions), **(B)** rats exposed to WIRS for 3.5 h (developed lesions), **(C)** omeprazole-treated, and **(D)** *P. sarmentosum*-treated. Arrows indicate gastric lesions.

**FIGURE 2:**
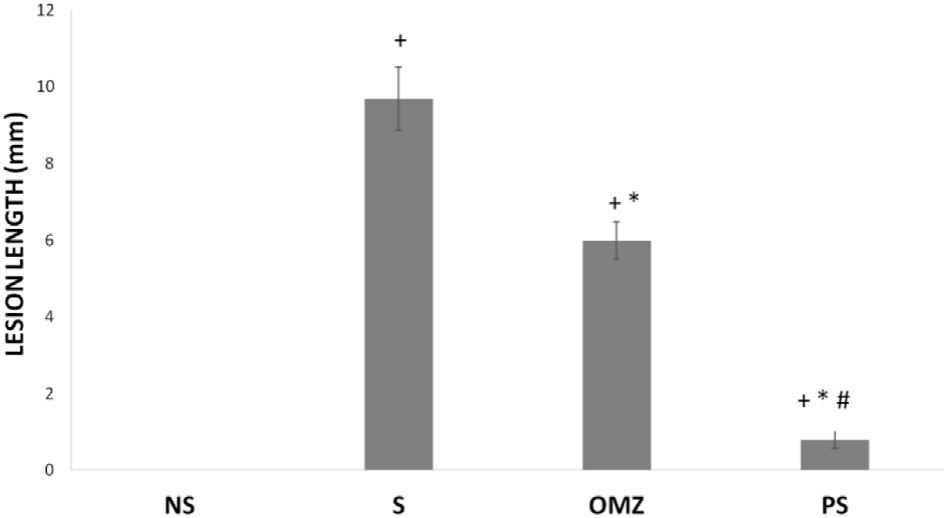
Effects of *P. sarmentosum* (500 mg/kg; PS) and omeprazole (20 mg/kg; OMZ) on gastric lesion length (in mm) in rats exposed to WIRS. Bars represent mean ± SEM (*n* = 7). Key: +, *p* < .05 compared to the non-stress (NS) control group; *, *p* < .05 compared to the stress (S) control group; #, *p* < .05 compared to the OMZ-treated group.

No lesions were observed in the stomachs of rats in the non-stressed control group. Rats exposed to WIRS for 3.5 h developed gastric lesions at the glandular part of the stomach (Figure 1). Supplementation with *P. sarmentosum* or omeprazole significantly lowered gastric lesion scores (*p* = .002 and *p* = .026, respectively; Figure 2). Moreover, *P. sarmentosum* reduced stomach lesions significantly more than omeprazole (*p* = .002; Figure 2).

### 3.2 Antioxidant levels

Gastric MDA content was significantly higher in stress-exposed rats than in non-stressed rats (*p* = .001; Table 2). *P. sarmentosum* and omeprazole supplementation attenuated the stress-induced increase in MDA levels, maintaining them at similar levels to non-stress rats. *P. sarmentosum* and omeprazole showed comparable effects on WIRS-induced gastric MDA levels. In addition, WIRS exposure significantly reduced gastric SOD activity (*p* = .002). However, *P. sarmentosum* and omeprazole *s*upplementation did not attenuate the reduction in SOD activity in stress-exposed rats.

**TABLE 2:** The effect of *P. sarmentosum* and omeprazole on MDA and NO levels and SOD activity in rats exposed to WIRS for 3.5 h.

Treatment	MDA content (mmol/tissue)	SOD activity (U/mg)	NO level (µM)
Non-stress control	1.725*	.6371	10.944
Stress control	4.656	.1173^+^	15.5^+^
Omeprazole	2.217*	.213^+^	14.207^+^
*Piper sarmentosum*	2.903*	.0882^+^	10.676*

+ vs. non-stress control (*p* < .05), * vs. stress control (*p* < .05), # vs. omeprazole (*p* < .05).

**FIGURE 3:**
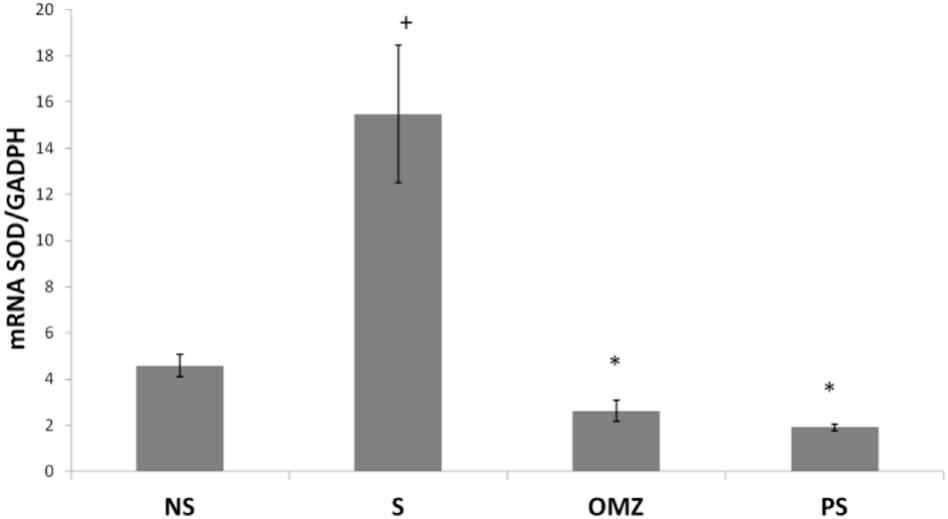
Effects of *P. sarmentosum* (500 mg/kg; PS) and omeprazole (20 mg/kg; OMZ) on gastric *Sod* mRNA levels in rats exposed to WIRS for 3.5 h. Bars represent mean ± SEM (*n* = 7). Key: +, *p* < .05 compared to the non-stress (NS) control group; *, *p* < .05 compared to the stress (S) control group; #, *p* < .05 compared to the omeprazole-treated group.

SOD mRNA levels were significantly lower in non-stressed rats than in stress-exposed rats. In addition, SOD mRNA levels were significantly lower in stress-exposed rats treated with *P. sarmentosum* or omeprazole than in untreated stress-exposed rats (Figure 3).

### 3.3 NO and iNOS mRNA levels

**FIGURE 4:**
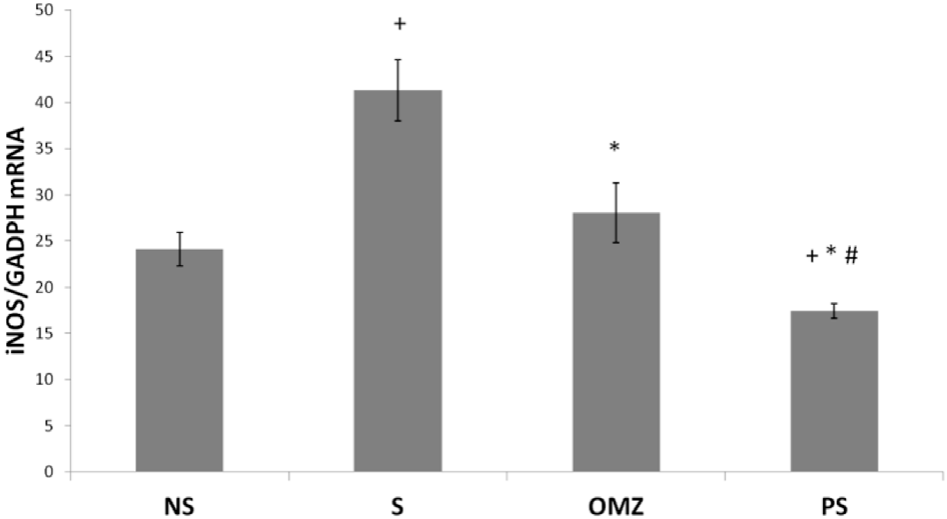
Effects of *P. sarmentosum* (500 mg/kg; PS) and omeprazole (20 mg/kg; OMZ) on gastric iNOS mRNA levels in rats exposed to WIRS for 3.5 h. Bars represent mean ± SEM (*n* = 7). Key: +, *p* < .05 compared to the non-stress (NS) control group; *, *p* < .05 compared to the stress (S) control group; #, *p* < .05 compared to the omeprazole-treated group.

NO levels were significantly higher in stress-exposed rats than in non-stressed rats (*p* = .036). *P. sarmentosum* but not omeprazole supplementation significantly attenuated the increase in NO levels (*p* = .026; Table 2). In addition, iNOS mRNA levels were significantly higher in stress-exposed rats than in non-stressed rats (Figure 4). However, *P. sarmentosum* and omeprazole supplementation significantly attenuated the stress-induced elevation in gastric iNOS mRNA levels. Moreover, iNOS mRNA levels were significantly lower with *P. sarmentosum* than with omeprazole supplementation.

### 3.4 Cytokines

**FIGURE 5:**
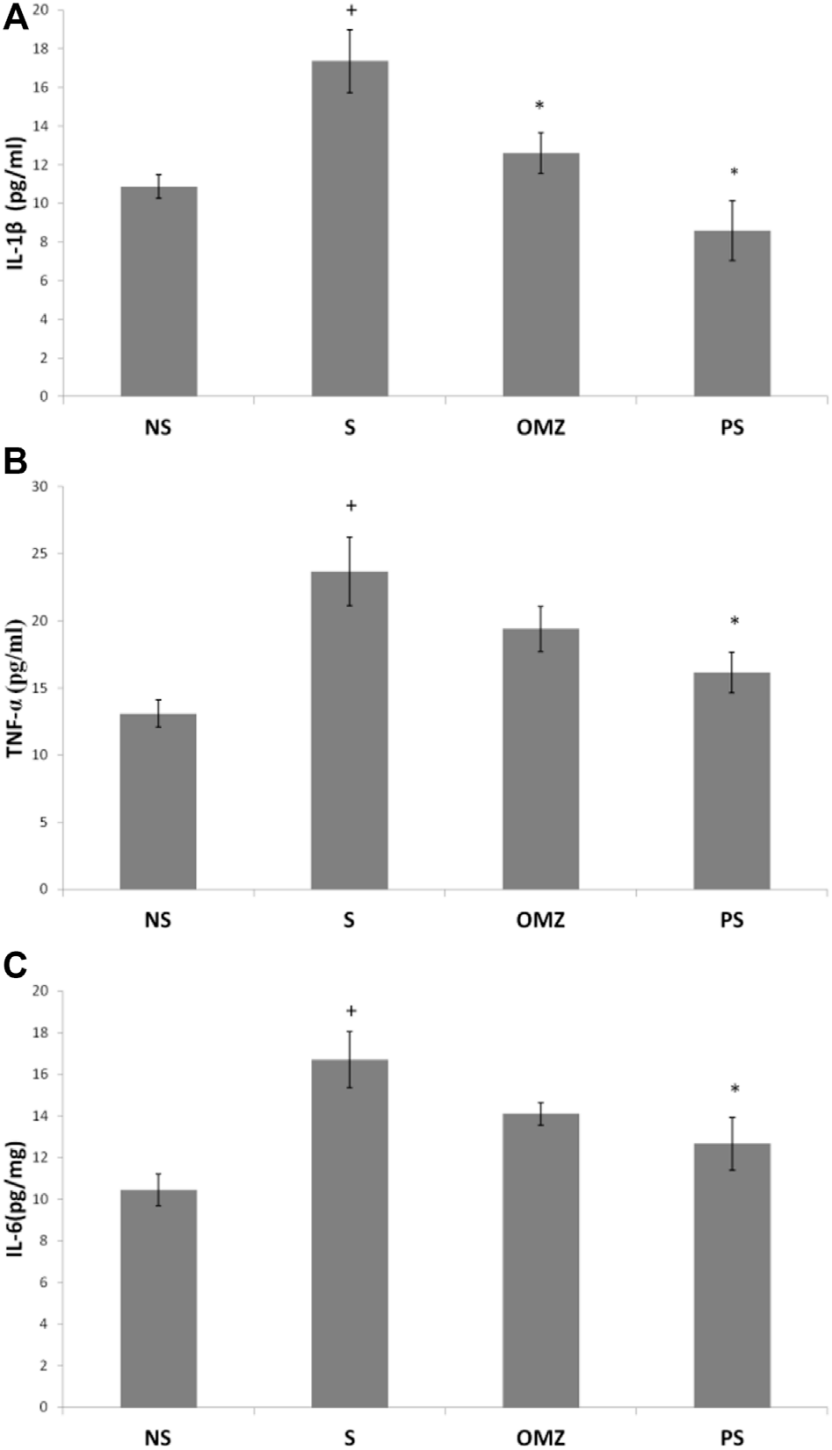
Effects of *P. sarmentosum* (500 mg/kg; PS) and omeprazole (20 mg/kg; OMZ) on gastric **(A)** TNF-α, **(B)** IL-1β, and **(C)** IL-6 protein levels in rats exposed to WIRS for 3.5 h. Bars represent mean ± SEM (*n* = 7). Key: +, *p* < .05 compared to the non-stress (NS) control group; *, *p* < .05 compared to the stress (S) control group; #, *p* < .05 compared to the omeprazole-treated group.

Gastric TNF-α levels were significantly higher (∼2-fold) in stress-exposed rats than in non-stressed rats (*p* = .001; Figure 5A). *P. sarmentosum* supplementation significantly attenuated the increase in TNF-α levels in stress-exposed rats (*p* = .026). However, omeprazole supplementation did not affect TNF-α levels.

In addition, gastric IL-1β levels were significantly increased in stress-exposed rats than in non-stressed rats (*p* = .004; Figure 5B). However, *P. sarmentosum* (*p* = 1.0 × 10^–4^) and omeprazole (*p* = .043) supplementation significantly attenuated the increase in IL1-β levels in stress-exposed rats. Gastric IL-1β levels did not differ significantly between stress-exposed rats given *P. sarmentosum* and omeprazole supplements, suggesting they have comparable beneficial effects.

Gastric IL-6 levels were significantly increased in stress-exposed rats than in non-stressed rats (*p* = .004; Figure 5C). *P. sarmentosum* supplementation significantly attenuated the increase in gastric IL-6 levels in stress-exposed rats (*p* = .044). However, omeprazole supplementation did not restore gastric tissue IL-6 levels to their non-stressed values (*p* = .043).

## 4 Discussion

Our findings showed that rats exposed to WIRS for 3.5 h developed gastric lesions at the glandular part of the stomach, consistent with previous studies (Konturek et al., 2008; Kwiecien et al., 2012; Azlina et al., 2017). This study showed that *P. sarmentosum* could protect gastric mucosa against stress-induced injury, supporting this plant’s use as a preventive treatment against gastric mucosa injury. *P. sarmentosum* supplementation provided better protection against stress-induced gastric ulcers than omeprazole. Omeprazole is an established gastric ulcer treatment used in numerous studies to provide gastroprotective effects (Hajrezaie et al., 2012; Sidahmed et al., 2013). However, this study showed that pretreatment with *P. sarmentosum* provided better protection in reducing gastric mucosal lesions than omeprazole. Our assessment of gastric lesions was based on measuring their greatest diameter and averaging their total length.

Chan et al. (2019) described *P. sarmentosum*’s ability to inhibit pro-inflammatory cytokines such IL-1β, TNF-α, and Il-6. Omeprazole affects cytokines differently from *P. sarmentosum,* decreasing IL-1β levels but increasing IL-6 and TNF-α levels compared to the non-stress control group. While studies in *H. pylori*-infected humans have shown omeprazole to positively affect pro-inflammatory cytokines (Kountouras et al., 2000), our rat-based study did not show similar effects. In addition, IL-1β significantly increased intercellular adhesion molecule 1 expression (Watanabe et al., 2001) and leucocyte infiltration in the scarred mucosa’s superficial region before ulcer recurrence (Watanabe et al., 1997). They discovered that inhibiting gastric acid sufficiently with omeprazole prevented both ulcer recurrence and responses, indicating that acid may enhance gastric mucosal inflammation in response to IL-1β stimulation, resulting in gastric ulcers. Metabolic pathway analysis indicated that *P. sarmentosum* exerts anti-inflammatory activity mainly by affecting tryptophan metabolism (Wang et al., 2020). Its metabolic product, melatonin, has anti-inflammatory properties by inhibiting the activation of nuclear factor kappa-light-chain-enhancer of activated B cells (Wang et al., 2020). These findings are consistent with our results showing *P. sarmentosum*’s anti-inflammatory activity on the inflammatory signaling pathway by downregulating IL-1β, IL-6, and TNF-α.

The enhanced expression and release of IL-1β, IL-6, and TNF-α could also contribute to increased reactive oxygen species production in the gastric mucosa. Lipid peroxidation arises in biological systems due to the oxidation of unsaturated, mostly polyunsaturated, lipids, leading to the formation of free radicals and lipid peroxides that are harmful to viable tissues. Lipid peroxides quickly decompose to produce many compounds. One common byproduct of that process is MDA. MDA is present in serum, plasma, and tissues because of lipid peroxidation. It is the most reported analyte for estimating lipid peroxidation and oxidative stress (Del Rio et al., 2005; Rappaport, 2006). Our results indicate that MDA levels are significantly higher in stress-exposed rats. This study confirms the involvement of free radicals in the pathogenesis of stress-induced gastric injuries. This finding is consistent with other studies showing the importance of lipid peroxidation in causing injuries to the gastric mucosa (Kamisah et al., 2011; Nur Azlina et al., 2013).

Antioxidants have been shown to protect against gastric mucosa injury (Nur Azlina et al., 2009; Ibrahim et al., 2012; Azlina et al., 2015). This study showed that *P. sarmentosum* significantly reduced MDA levels compared to stress control rats, which probably reduced gastric injury by retarding the lipid peroxidation process. *P. sarmentosum* has higher antioxidant activity than other traditional plants (Chanwitheesuk et al., 2005). This plant also contains a natural antioxidant (naringenin, didymin, methyl piperate, quercetin, beta asarone, brachyamide, amurensin, piperitol, guineensine, hesperidin, rutin, malvidin, and difucol) (Subramaniam et al., 2003; Bactiar and Fahami, 2019; Raja Kumar et al., 2019), which might contribute to *P. sarmentosum*’s ability to reduce MDA. Our findings also showed no differences between *P. sarmentosum* and omeprazole in reducing gastric MDA levels, suggesting they have similar radical scavenging abilities. Omeprazole was previously shown to confer dose-dependent protection against ethanol (Sener et al., 2004) and stress-induced (Azlina et al., 2015) acute gastric mucosal injury by inhibiting lipid peroxidation.

Several reactive oxygen species scavenging systems, including SOD, glutathione peroxidases, and catalases, prevent their destructive action. SOD catalyzes the dismutation of O_2_-into less noxious hydrogen peroxide, which is further degraded by catalases or glutathione peroxidases. This study found that stress exposure led to lipid peroxide production, indicated by increased gastric tissue MDA levels and SOD mRNA levels. This effect could be due to the normal physiological response upregulating SOD expression due to impaired antioxidative enzymes. Dinu et al. (2009) found reduced SOD activity in the gastric mucosa due to stress, increasing lipid peroxidation. *P. sarmentosum* and omeprazole supplementation did not increase SOD expression and enzyme activity, suggesting that they do not affect SOD activity. Reduced MDA levels and lesion occurrences in the *P. sarmentosum*-supplemented group indicate that it acts as an antioxidant (Subramaniam et al., 2003), potentially reflecting its naringenin content, a natural antioxidant superoxide scavenger (Bactiar and Fahami, 2019; Raja Kumar et al., 2019). Antioxidant agents have been shown to be effective in treating gastric ulcers (Kudryavtsev et al., 2014).

NO exerts either protective or destructive effects depending on the extent of its production. While NO produced by endothelial NO synthase plays an important role in gastric ulcer formation and healing, NO produced by iNOS only participates in ulcer formation. This study has shown that iNOS mRNA levels were significantly higher in stress-exposed rats than in non-stressed control rats, with a concomitant increase in NO levels in the stress-exposed rats. This change is mainly due to excessive iNOS NO production in inflammatory cells, inducing oxidative tissue stress and promoting mucosal damage (Cho, 2001; Mittal et al., 2014). Nitrite levels were significantly elevated, potentially due to iNOS stimulation, which reacts with superoxide to form peroxynitrite, a potent cytotoxic oxidant causing gastric tissue damage (Lanas, 2008; Azlina et al., 2015). *P. sarmentosum s*upplementation significantly reduced stress-induced increases in NO levels and iNOS expression*.* It also reduced iNOS expression to a significantly greater extent than omeprazole. Therefore, its beneficial effects are likely due to its ability to decrease gastric iNOS expression and NO levels, reducing gastric mucosal damage.

## 5 Conclusion

Our findings provide evidence that oral *P. sarmentosum* supplementation confers protection against stress-induced gastric lesions, possibly *via* its antioxidant mechanism, reducing pro-inflammatory cytokines, and its effects on NO through reduced iNOS expression. *P. sarmentosum* extract showed a better protective effect than omeprazole in reducing gastric lesions; TNF-α, IL-6, NO levels; and iNOS expression. Therefore, it is a potential therapeutic agent for gastric ulcers with similar pathologies.

## Data Availability

The original contributions presented in the study are included in the article/supplementary material, further inquiries can be directed to the corresponding author.

## Abbreviations

4-HNE, 4-hydroxynonenal; eNOS, endothelial NO synthase; FRIM, Forest Research Institute Malaysia; H_2_O_2_, hydrogen peroxide; iNOS, inducible NO synthase; IL-1β, interleukin-1β; MDA, malondialdehyde; NO, nitric oxide; NSAIDS, non-steroidal anti-inflammatory drugs; O_2_•^-^, radical anions; OMZ, omeprazole; PS, *Piper sarmentosum*; SAPE, streptavidin phycoerythrin; SOD, superoxide dismutase; SOD, superoxide dismutase enzyme; TNF-α, tumor necrosis factor-α; UKMAEC, University Kebangsaan Malaysia Animal Ethics Committee; WIRS, water-immersion restraint stress.
